# The risk factors for miscarriage of viable intrauterine pregnancies in patients with heterotopic pregnancy after surgical intervention

**DOI:** 10.1097/MD.0000000000036753

**Published:** 2023-12-22

**Authors:** Heng-chao Ruan, Yan-hua Zhang, Lu Chen, Wei-xiao Zhou, Jun Lin, Hong Wen

**Affiliations:** a Department of Gynecological Oncology, Women’s Hospital, Zhejiang University School of Medicine, Hangzhou, China.

**Keywords:** heterotopic pregnancy, miscarriage, outcome, risk factor, surgical intervention

## Abstract

To summarize the clinical characteristics and explore the risk factors for miscarriage of a viable intrauterine pregnancy following surgical intervention in patients with heterotopic pregnancy (HP). A total of 106 women diagnosed with HP that underwent surgical intervention in the Women’s Hospital School of Medicine Zhejiang University between January 2014 and December 2021 were included in this retrospective study. They were divided into a miscarriage group (n = 13) and an ongoing pregnancy group (n = 93) according to the outcomes of the HP within 2 weeks after surgery. Data regarding clinical characteristics, surgical conditions, postoperative recovery, and complications were collected and compared between the groups. Logistic multivariate analysis was performed to explore the risk factors for miscarriage in patients with HP within 2 weeks of surgical intervention. Among the 106 women with HP, 80 had tubal HP, 8 had cornual HP, and 18 had interstitial HP. Eighty-seven (82.1%) patients developed clinical symptoms that manifested primarily as abnormal vaginal bleeding and/or abdominal pain, whereas 19 (17.9%) patients had no clinical symptoms. The mean gestational age on the day of surgery was 7.2 weeks (inter-quartile range, 6.4–8.3). The miscarriage rate within 2 weeks of surgical intervention was 12.3% in patients with HP. Compared to the ongoing pregnancy group, the miscarriage group had a higher body mass index, earlier gestational age at treatment, and higher volume of hemoperitoneum (*P* < .05 for all). Logistic multivariate analysis indicated that the women with a hemoperitoneum volume > 200 mL had significantly higher risk of miscarriage after adjusting covariates [OR (odds ratio) = 5.285, 95% CI (confidence interval) (1.152–24.238), *P < *.05]. Hemoperitoneum volume was independently associated with miscarriage of viable intrauterine pregnancies in patients with HP within 2 weeks of surgical intervention.

## 1. Introduction

Heterotopic pregnancy (HP) is defined as the coexistence of intrauterine pregnancy (IUP) and ectopic pregnancy (EP), which is a rare condition with an incidence of approximately 1 per 30,000 pregnancies.^[1,2]^However, with the increased application of assisted reproductive techniques (ART) such as in vitro fertilization and artificial insemination (AI), the incidence of HP in a woman with ART treatment has raised to approximately 1%.^[3–5]^ It is essential to diagnose and treat HP as soon as possible to avoid severe complications, such as hypovolemic shock, emergency blood transfusions, maternal mortality, and fetal loss. The early and accurate diagnosis of HP remains challenging due to the presence of IUP. As a result, identifying the risk factors of HP as early as possible is crucial to optimize diagnosis.

The primary goal of treating HP is to remove the ectopic pregnancy while maintaining intrauterine pregnancy.^[6]^ Treatment for HP includes expectant management, embryo aspiration with or without embryo-killing drugs, and surgical intervention. Severe morbidity and mortality in pregnant women can be reduced through early diagnosis and proper management of HP. The primary surgical intervention for HP involves laparoscopic or laparotomic surgery to remove the EP. However, few studies have investigated the risk factors for postoperative HP miscarriage. Consequently, it is important to explore the risk factors of IUP miscarriage in patients with HP after surgery.

In the present study, we described the clinical characteristics of HP in a single medical center and investigated the risk factors for miscarriage in viable intrauterine pregnancies within 2 weeks after surgery.

## 2. Materials and methods

### 2.1. Study procedure and sample

**Figure 1.**
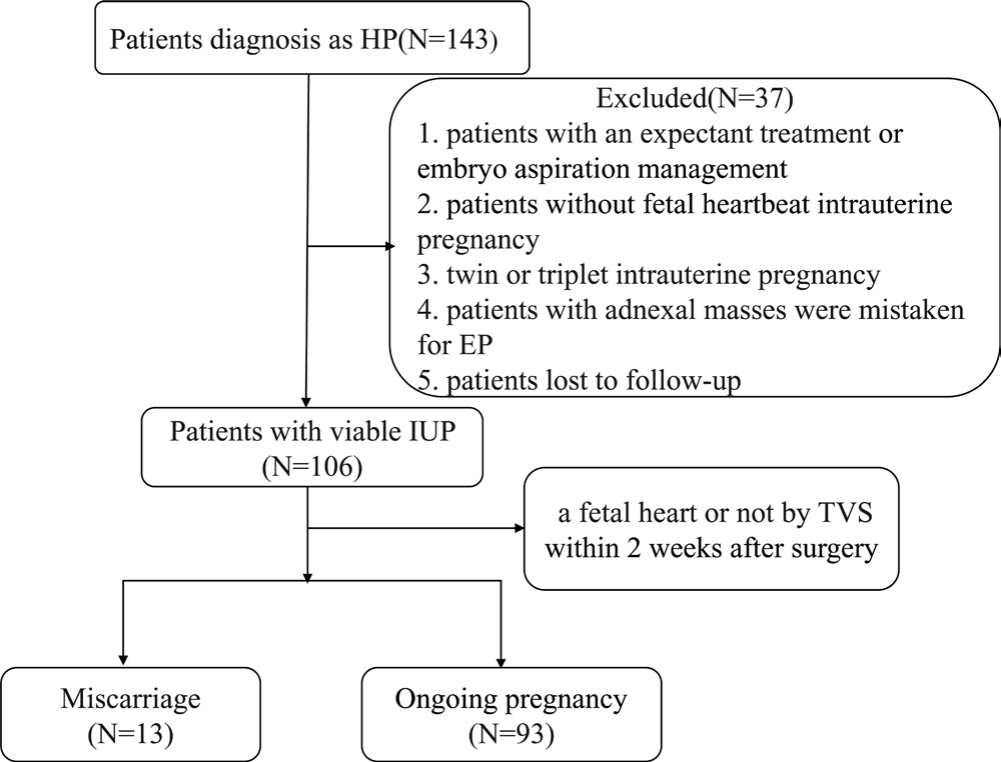
Flow diagram of study.

This retrospective study initially included 143 patients that were preoperatively diagnosed with HP at the Women’s Hospital School of Medicine, Zhejiang University, between January 2014 and December 2021. The inclusion criterion consisted of HP diagnosed through ultrasound, which was based on the visualization of an intrauterine fetal heartbeat concomitant with an ectopic pregnancy. Ultrasounds were performed by at least two sonographers to reduce the likelihood of misdiagnoses. Patients who met one of the following criteria were excluded: expectant treatment or embryo aspiration management, no fetal heartbeat during intrauterine pregnancy, twin or triplet intrauterine pregnancy, adnexal masses mistaken for EP, and lost during follow-up (Fig. 1). All patients underwent surgical intervention, including laparotomy or laparoscopy depending on their condition and willingness to undergo surgery. In comparison to open surgery, laparoscopic skills primarily involve establishing pneumoperitoneum and administering general anesthesia. Additionally, a pathological examination was conducted for all patients following surgical intervention to confirm the diagnosis definitively. Finally, the clinical data of 106 patients who met the inclusion criteria for surgically and pathologically confirmed HP were collected and analyzed retrospectively. All the patients received progesterone supplements after surgical intervention, and transvaginal ultrasonography was routinely performed on patients in the 1st and 2nd weeks after surgery. Based on transvaginal ultrasonography and clinical features, the patients were divided into a miscarriage group (n = 13) and an ongoing pregnancy group (n = 93).

Human experimentation procedures were conducted in accordance with the Helsinki Declaration of 1975, revised in 2008, and with the ethical standards of the responsible committee for human experimentation. This study was approved by the Institutional Review Board of our hospital (approval number: IRB-20220289-R). The requirement for informed consent was waived because personal information was anonymized.

### 2.2. Data collection

Based on medical records, we retrospectively collected data on each patient’s age, body mass index, previous ectopic pregnancy, previous gynecological surgery, mode of conception, heterotopic pregnancy type, and gestational age at treatment.

Data on the surgical intervention were collected from each patient, including treatment method, operative time, and volume of intraoperative hemorrhage.

Ectopic pregnancy refers to pregnancies that occur partially or completely outside the uterine cavity, including pregnancies that occur in the ovaries, fallopian tubes, cesarean scar, or cervix. A viable IUP was defined as fetal heartbeats of the IUP visible on transvaginal ultrasonography examination. In this study, the operation time was defined as the time between incision and closure of the skin. Miscarriages subsequent to treatment were defined as abortions occurring prior to the completion of 12 weeks of gestation.^[7]^ Clinical characteristics including maternal age, history of ectopic pregnancy, body mass index, pelvic surgery, gestational weeks at diagnosis and operative day, treatment method, clinical manifestations, operative time, and obstetric outcomes were reviewed for analysis.

### 2.3. Statistical analysis

All statistical analyses were performed using SPSS 20.0. Continuous variables were expressed as mean ± SD or inter-quartile range (IQR) depending on their normality. Categorical variables are expressed as percentages (numbers). During the analysis, continuous variables were analyzed with the Student *t* test, and categorical variables were analyzed with the Chi-square test or Fisher exact test. The logistic regression analysis was conducted to explore the independent risk factors for miscarriage in patients with HP within 2 weeks after surgery and the results were expressed as odds ratios (ORs) with 95% confidence intervals (CIs). Statistical significance was set at < 0.05.

## 3. Results

### 3.1. The clinical characteristics of women with HP

In total, 106 women with HP were included in this study. The baseline clinical characteristics of patients with HP are shown in Table 1. Among the 106 patients, 13 (12.3%) became pregnant naturally and 93 (87.7%) became pregnant via ART treatment. The median age at admission was 30 years (IQR, 28–33). Sixty-eight (64.2%) women had a history of gynecological surgery and 27 (25.5%) patients had a history of EP. Eighty-seven (82.1%) patients developed clinical symptoms that manifested primarily as abnormal vaginal bleeding and/or abdominal pain, whereas 19 (17.9%) patients had no clinical symptoms. As to the location of the EP, 80 (75.5%) patients had tubal pregnancies and 26 (24.5%) patients had interstitial or cornual pregnancies, as determined by TVs and subsequent confirmation via laparoscopy or laparotomy.

**Table 1:** Comparison of clinical characteristics of patients with HP.

Variables	All (n = 106)	Miscarriage group (n = 13)	Ongoing pregnancy group (n = 93)	Z/χ^2^	*P*
Age (yr)	30 (28, 33)	30 (27, 35)	30 (28, 33)	0.111	.912
Body mass index (kg/m^2^)	21.3 (19.3, 23.3)	23.3 (21.3, 24.1)	20.8 (19.1, 22.9)	2.350	.019
Previous ectopic pregnancy	27 (25.5)	4 (30.8)	23 (24.7)	0.533	.594
Previous gynecological surgery	69 (65.1)	7 (53.8)	62 (66.7)	1.222	.222
The mode of conception					
IVF	86 (81.1)	10 (76.9)	76 (81.7)		.915
AI	7 (6.6)	1 (7.7)	6 (6.5)		
Natural pregnancy	13 (12.3)	2 (15.4)	11 (11.8)		
Treatment method					
Laparoscopy	92 (86.8)	9 (69.2)	83 (89.2)	2.432	.119
Laparotomy	14 (13.2)	4 (30.8)	10 (10.8)		
Heterotopic pregnancy type					
Tubal pregnancy	80 (75.5)	8 (61.5)	72 (77.4)	2.315	.316
Cornual pregnancy	8 (7.5)	4 (30.8)	7 (7.5)		
Interstitial pregnancy	18 (17.0)	1 (7.7)	14 (15.1)		
Gestational age at treatment (wk)	7.2 (6.4, 8.3)	6.4 (5.9, 7.5)	7.4 (6.6, 8.4)	2.024	.043
Operation time (min)	50 (39, 65)	55 (40, 68)	50 (38, 65)	0.381	.703
Volume of hemoperitoneum (mL)					
≤ 200	67 (63.2)	4 (30.8)	63 (67.7)	5.209	.022
> 200	39 (36.8)	9 (69.2)	30 (32.3)		

Variables were presented as median (interquartile range) or number (%).

AI = artificial insemination, HP = heterotopic pregnancy, IVF = in vitro fertilization.

The median gestational age at diagnosis was 7.0 weeks (IQR, 6.1–8.0), while the median gestational age on the day of surgery was 7.2 weeks (IQR, 6.4–8.3).

### 3.2. Surgical characteristics of HP

The median operative time was 50 minutes (IQR, 39–65), and the estimated blood loss was 20 mL (IQR, 10–30). Hemoperitoneum (presence of blood in the peritoneal cavity) was detected in 79 of 106 (74.5%) patients. The volume ranged from 10 to 2000 mL, and in 9 (8.5%) patients was more than 1000 mL. All the patients were successfully treated without adverse maternal complications.

### 3.3. Comparisons of variables between the 2 groups

The results of the univariate regression analysis are presented in Table 1. The body mass index in the miscarriage group was significantly greater than that in the ongoing pregnancy group (23.3 vs 20.8, *P* = .019). Similarly, the gestational age at treatment was earlier in the miscarriage group than in the others (6.4 vs 7.4, *P* = .043). The proportion of women with a hemoperitoneum volume exceeding 200 mL was significantly higher in the miscarriage group (69.2%) compared to the ongoing pregnancy group (67.7%), with a *P* value of .022. However, there were no differences between the two groups in terms of age, previous ectopic pregnancy or gynecological surgery, heterotopic pregnancy type, mode of conception, treatment method, or operative time.

### 3.4. Logistic analysis of the risk factors for miscarriage of viable IUP after surgery

We further performed a multivariate logistic analysis of the variables that were statistically significant in the univariate analysis and could be predetermined as traditional risk factors. After adjusting covariates, multivariate analysis revealed that a hemoperitoneum volume >200 mL was an independent risk factor that was significantly related to the miscarriage of a viable IUP [OR = 5.285, 95% CI (1.152–24.238)] (Table 2).

**Table 2:** Multivariable logistic regression of miscarriage in patients with HP.

	OR (95% CI)	*P*
Age (yr)	0.953 (0.806–1.126)	.569
Body mass index (kg/m^2^)	1.224 (0.987–1.519)	.066
Heterotopic pregnancy type		
Tubal pregnancy	Ref.	.564
Cornual pregnancy	0.388 (0.023–6.466)	.510
Interstitial pregnancy	1.820 (0.367–9.023)	.464
Gestational age at treatment (wk)	0.689 (0.391–1.214)	.198
Operation time (min)	0.988 (0.947–1.031)	.582
Treatment method		
Laparotomy	Ref.	.169
Laparoscopy	0.092 (0.021–1.340)	
Volume of hemoperitoneum (mL)		
≤ 200	Ref.	.032
> 200	5.285 (1.152–24.238)	

HP = heterotopic pregnancy.

## 4. Discussion

The incidence of HP has increased dramatically with the widespread use of ART. The principal treatment for HP is the safe removal of the ectopic pregnancy while maintaining intrauterine pregnancy. However, establishing a standard or principal treatment for HP is difficult because of the characteristics and the rarity of its occurrence.

In contrast to laparotomy, carbon dioxide (CO2) pneumoperitoneum is used during laparoscopic surgeries. In theory, pneumoperitoneum decreases uterine blood flow and elevates maternal PaCO2, resulting in reduced transplacental fetal CO2 excretion and inhibiting the progression of respiratory acidosis. Despite this, one study found that maternal PaCO2, pH, umbilical artery pulsatility index, and uterine artery resistance index did not change significantly during laparoscopic surgery.^[8–10]^ In our study, those who underwent laparotomy had a significantly higher miscarriage rate than those who had laparoscopic surgery (28.7% vs 9.78%). However, the difference was not statistically significant. Laparoscopic surgery offers several advantages over laparotomy, including minimal invasiveness, short operation time, and rapid recovery time. Considering the situation described above, we suggest that laparoscopic management should be offered when the patient’s hemodynamics are stable. As most patients with HP conceive via ART, they often have higher expectations for IUP. In the present study, we conducted a subgroup analysis to explore the potential risk factors for miscarriage in viable IUPs within 2 weeks of surgery. Evidence suggests that earlier symptoms such as vaginal bleeding, and abdominal pain, along with a younger gestational age on the day of surgery are independent predictors of intrauterine pregnancy loss.^[1,4,11–13]^ However, we found that the signs or symptoms had no effect on the prognosis of viable IUP in patients with HP within 2 weeks of surgery. Additionally, we identified the hemoperitoneum volume as an independent predictor of miscarriage of viable IUP in patients with HP treated surgically. Although the underlying mechanisms are unclear, we speculate that hemoperitoneum may cause miscarriage due to irritation of the uterus, which may stimulate uterine contractions and subsequently lead to miscarriage.

Previous studies on HP have reported widely different rates of miscarriage rates of IUP, ranging from 14.1% to 33%.^[3,4,12,14]^ Possibly, this is because of the heterogeneity among definitions of pregnancy viability. Several studies have not distinguished between the outcomes of intrauterine pregnancies that were alive and those that were not alive at the time of diagnosis. In the present study, to minimize confounding factors, patients with intrauterine pregnancies of uncertain viability were excluded, such as those with a crown-rump length of < 7 mm, no heartbeat, mean sac diameter of < 25 mm, and no embryo shown by transvaginal sonography.^[15,16]^

A growing body of literature has demonstrated that treating the extrauterine pregnancy via invasive surgery has no effect on the risk of miscarriage of the concomitant alive IUP.^[17–20]^ Our study showed that the average pregnancy loss rate for HP within 2 weeks of surgery was 12.3% (13/106), which was similar to the 12% to 17% miscarriage rate reported in previous studies.^[1,11,13,21,22]^ Moreover, considering that spontaneous abortion occurs in 11% to 65% of viable IUPs, surgical intervention for HP is considered safe and does not augment the risk factors for miscarriage of viable IUPs in patients with HP.^[23]^

Our study has some limitations. First, only risk factors associated with general clinical characteristics and surgery were considered; other factors such as embryo quality, endometrial receptivity, and luteal support were not considered. Second, the sample size was small owing to the rarity of the disease in the present study. However, we believe that these data will provide valuable information for surgical intervention of HP in the future, and further studies involving larger sample sizes are needed to validate its safety and efficacy.

In conclusion, surgical treatment of HP with a viable IUP is a safe treatment option and does not appear to influence the rate of early pregnancy loss within 2 weeks after surgery. The hemoperitoneum volume may be a meaningful risk factor for predicting miscarriage of viable IUP in patients with HP within 2 weeks after surgery. Further studies are needed to identify the factors that influence pregnancy outcomes of viable IUP in patients with HP.

## Author contributions

**Conceptualization:** Heng chao Ruan, Yan-hua Zhang.

**Data curation:** Heng chao Ruan, Yan-hua Zhang.

**Formal analysis:** Heng chao Ruan, Yan-hua Zhang.

**Investigation:** Lu Chen.

**Methodology:** Wei-xiao Zhou.

**Project administration:** Jun Lin.

**Resources:** Hong Wen.

**Software:** Heng chao Ruan.

**Supervision:** Hong Wen.

**Writing – original draft:** Heng chao Ruan, Yan-hua Zhang.

**Writing – review & editing:** Hong Wen.
